# Protective Roles of Honey in Reproductive Health: A Review

**DOI:** 10.3390/molecules26113322

**Published:** 2021-06-01

**Authors:** Siti Sarah Mohamad Zaid, Siti Suraya Ruslee, Mohd Helmy Mokhtar

**Affiliations:** 1 Department of Environment, Faculty of Forestry and Environment, Universiti Putra Malaysia, Serdang 43400, Malaysia; surayaruslee08@gmail.com; 2 Department of Physiology, Faculty of Medicine, Universiti Kebangsaan Malaysia, Kuala Lumpur 56000, Malaysia; helmy@ukm.edu.my

**Keywords:** honey, reproductive health, therapeutics, fertility

## Abstract

Nowadays, most people who lead healthy lifestyles tend to use natural products as supplements, complementary medicine or alternative treatments. Honey is God’s precious gift to mankind. Honey has been highly appreciated and extensively used since ancient history due to its high nutritional and therapeutic values. It is also known to enhance fertility. In the last few decades, the important role of honey in modern medicine has been acknowledged due to the large body of convincing evidence derived from extensive laboratory studies and clinical investigations. Honey has a highly complex chemical and biological composition that consists of various essential bioactive compounds, enzymes, amino and organic acids, acid phosphorylase, phytochemicals, carotenoid-like substances, vitamins and minerals. Reproductive health and fertility rates have declined in the last 30 years. Therefore, this review aimed to highlight the protective role of honey as a potential therapeutic in maintaining reproductive health. The main role of honey is to enhance fertility and treat infertility problems by acting as an alternative to hormone replacement therapy for protecting the vagina and uterus from atrophy, protecting against the toxic effects of xeno-oestrogenic agents on female reproductive functions and helping in the treatment of gynaecological disorders, such as vulvovaginal candidiasis infection, that affect women’s lives.

## 1. Introduction

Honey is a supersaturated aqueous solution of inverted sugar. It mainly comprises 38% fructose, 31% glucose and a small amount of a complex mixture of disaccharides, trisaccharides, maltose, maltulose and oligosaccharides. It contains a wide variety of essential bioactive compounds, as well as various types of enzymes, including amino and organic acids, acid phosphorylase, phytochemicals, carotenoid-like substances, vitamins and minerals [1,2]. It also contains a trace amount of vitamin A (retinol), vitamin E (tocopherol), vitamin K (an antihaemorrhagic vitamin), vitamin B1 (thiamine 1), vitamin B6, vitamin C (ascorbic acid), niacin, folic acid, minerals and iron zinc [3].

Honey is a suitable supplement for humans due to its high nutritional value (304 kcal/100 g honey) and its rapidly absorbable carbohydrate content. It has a density of approximately 1.36 kg/L and is 40% denser than water; it also presents an acidic environment with pH levels ranging from 3.2 to 4.5, high osmotic pressure and low water content [4]. Raw honey is usually produced in small farms without undergoing specific processes, such as thermal treatment or pasteurisation, to alter its natural composition. However, the extraneous matter is removed from honey to make it marketable on a large scale [5].

The main enzymes present in honey are invertase, diastase (amylase), catalase, glucose oxidase and acid phosphatase [6]. It also comprises several amino acids, such as proline, lysine, phenylalanine, tyrosine, glutamic and aspartic acids, that are produced by pollen, nectar or honeybees. Gluconic acid is the predominant organic acid in honey, followed by other types of acids, such as butyric, acetic, formic, lactic, succinic, malic, citric, maleic, oxalic and pyroglutamic acids [7]. Gluconic acid in honey mainly originates from enzymatic glucose oxidation. Notably, gluconic acid confers a slightly tart flavour, antimicrobial properties and enhanced calcium absorption to honey.

**Figure 1:**
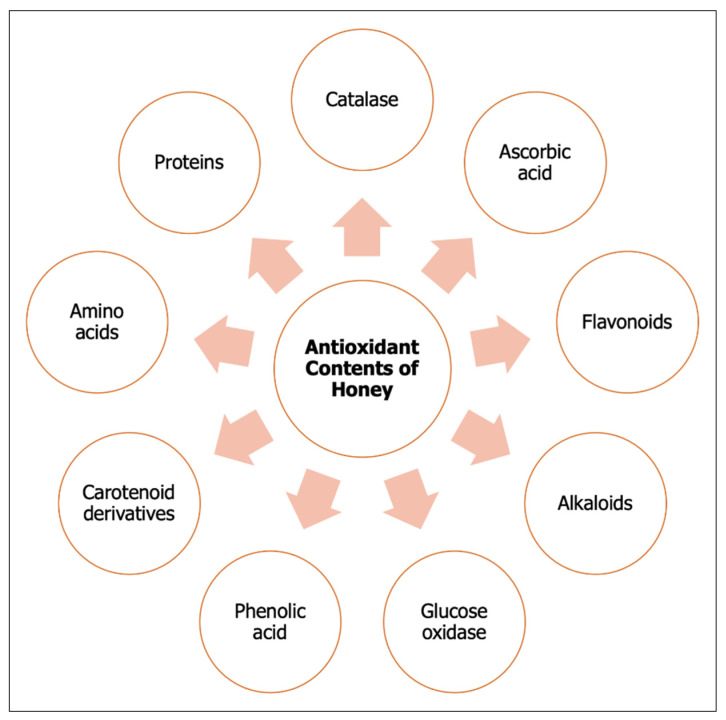
Antioxidant contents of honey.

Phytochemicals are non-nutrient compounds that are commonly found in plants, such as fruits and vegetables [8]. They offer health-promoting activities by reducing the risk of oxidative tissue [9,10]. Honey is known to be rich in enzymatic and nonenzymatic antioxidants, including catalase, ascorbic acid, flavonoids, alkaloids, glucose oxidase, phenolics acid, carotenoid derivatives, Maillard reaction products, amino acids and proteins, as shown in Figure 1 [9,10,11]. A unique flavonoid, known as pinocembrin, is present in propolis and honey; other types of flavonoids, including quercetin, chrysin, galangin, luteolin and kaempferol, are also found in honey [12]. The presence of these bioactive compounds contributes to the fundamental knowledge necessary to investigate the possible physiological roles of honey in health promotion.

The use of natural food as nutraceuticals to treat illnesses and diseases has received considerable attention in recent years. Additionally, the mechanism of the health effects of honey has gradually become apparent in numerous studies. Many researchers have demonstrated that honey is a rich source of natural antioxidants and phytochemicals and thus effectively reduces the risk of heart disease, cancer, immune system decline, cataracts and inflammatory processes [13,14]. It also acts as an antiallergic, antithrombotic and vasodilatory agent while exerting positive effects on gastrointestinal health and energy metabolism [15].

Many studies have also demonstrated the functions of honey in health maintenance and reproductive functions. With respect to female reproductive health, honey is capable of preventing uterine and vaginal atrophy and dryness in a menopausal animal model [16,17]. Moreover, the toxic effects of BPA on follicular development could be reduced by honey intake [18,19]. A clinical study found that honey was an effective treatment for vulvovaginal candidiasis (VVC) infections [20]. Honey is mainly used with egg yolk by local folk in Asia during the postpartum and postmenopausal periods to improve health and enhance vitality. Royal jelly and propolis are other beehive products that have been scientifically proven to improve menopausal syndromes [21,22]. For male reproductive health, honey was found to protect rat testes from oxidative stress [23] and maintain sexual behaviour [24] in cigarette smoke (CS)-exposed rats. It also preserved the quality of sperm when used in semen extenders and served as a spermiogenesis booster [24,25]. Accordingly, this review aims to highlight the protective roles of honey as a potential therapeutic in the maintenance of reproductive health.

## 2. Production and Variations in Honey

International honey production is on the rise, owing largely to Asia, especially China and India, which have seen average annual increases in honey production of more than 10,000 tonnes. This could meet the market demand, which is expanding every year. Besides that, the growth of technological and scientific advancement contributed to rapid expansion in honey production, resulting in a total of 1.8 million tonnes of honey being produced in 2016 worldwide [26].

In term of variations, previous studies indicate that the composition of honey varies widely in accordance with the floral source and geographical origin, climate, environmental conditions and postharvest procedures, such as processing, handling and storage [27,28]. Approximately more than 300 types of honey originate from the various flowers and plants that are consumed by honeybees. Italian honey collected from the nectar of wild blossoms in hill and mountain zones exhibits strong organoleptic characteristics, high nutritional values and density and low moisture content [29]. It also presents stability, which guarantees a long shelf life without the risk of fermentation or changes in properties [5].

Multifloral honey is different from unifloral honey in the sense that it is darker in colour. Although it undergoes solid crystallisation and has a thick texture, unifloral honey has a light colour, transparent appearance and thin texture [5]. Honey colour is influenced by the mineral, pollen and phenolic contents of the honey [12,30]. Honey may darken during storage due to Maillard reactions, fructose caramelisation, polyphenol reactions, temperature and storage time [15].

Previous studies have shown a strong correlation between honey colour and antioxidant power. Specifically, honey with a dark colour and high crystallisation has a high total phenolic content and thus has a stronger antioxidant capacity than light and transparent honey [5,15,31]. Moreover, honey colour is dependent on potential alkalinity and ash content, as well as the presence of antioxidative active pigments, including carotenoids and flavonoids [32]. In addition, differences in honey composition can influence the value of specific honey types for medicinal or health-promoting purposes [2,33]. Nevertheless, the main constituents of all honey types from all countries around the world are almost similar.

## 3. Honey and Reproductive Health

**Figure 2:**
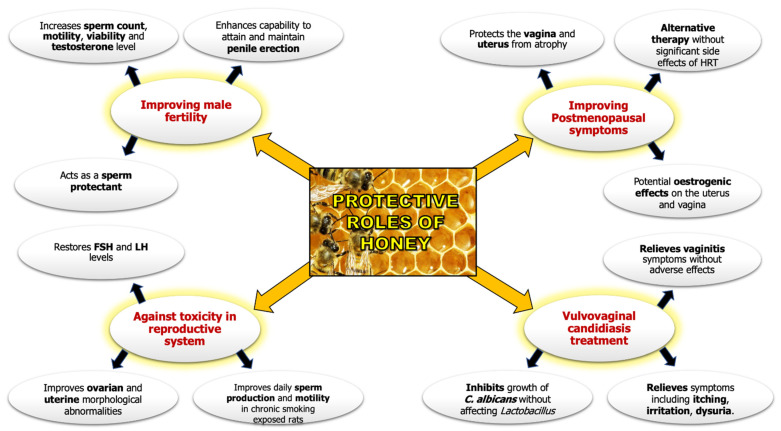
Protective roles of honey in reproductive health.

Historically, honey is traditionally used in many cultures to enhance fertility in women and men. The adaptation of traditional practices remains relevant amongst complementary and alternative medicine practitioners. In general, honey has been acknowledged for its vasodilation effects that improve erections in men who face impotence issues. Furthermore, in traditional practices, the routine intake of honey in milk is believed to considerably improve sperm counts and testosterone concentrations. In Malaysia, honey is extensively used by traditional practitioners as an efficacious ingredient for the production of the nutraceutical products *maajun* and *jamu*. These products are used to strengthen the reproductive tract (vagina and uterus) and improve egg quality. Figure 2 shows the protective roles of honey in reproductive health.

### 3.1. Role of Honey in Male Fertility

The 2015 data from the Population Division of the United Nations state that the fertility rates of most populations of developing countries have been decreasing for the past few years. In Malaysia, the fertility rate has dropped to more than half, and the birth rate has significantly dropped from six to two children per woman [34]. Moreover, male reproductive health and fertility rates have been declining for the last 30 years [35]. Younglai et al. attributed this unhealthy trend to society’s modern lifestyle, including smoking and routine exposure to various toxins through food, beverages and polluted environments [36].

In the last few decades, researchers in the field of complementary and alternative medicine have focused on the use of natural or herbal products to improve and treat fertility-related issues. Historically, the Egyptian community has practised the traditional use of honey as one of the potential remedies for infertility and vitality enhancement [37]. Many cultures believe that the routine intake of warm milk and honey is effective in considerably increasing sperm counts in subfertile or infertile men. Interestingly, the traditional Malay community uses a mixture of honey and eggs as a massage oil to treat erectile dysfunction (ED) [38].

Mohamed et al. (2012) treated rats that had been exposed to cigarette smoke (CS) with the daily oral supplementation of honey at a dose of 1.2 g/kg [25]. In contrast to the rats that were exposed only to CS, the rats fed with honey showed positive results in erectile function. The rats’ capability to attain and maintain penile erection was also enhanced, consequently improving sexual behaviours. These findings suggested that honey could be used against the adverse effects of CS in male rats and assist in treating ED.

Reductions in testosterone levels along with aging are commonly related to erection problems. The gradual decline in the level of testosterone as men age can be associated with ED issues. In male rats, the administration of 10% honey increased testosterone levels [39]. In a recent study, male rats fed with Persian honey after ischaemia–reperfusion-induced testis injury showed significant improvements as reflected by increases in sperm counts and testosterone levels [40].

The oral supplementation of 5% Palestinian honey for 20 days increased epididymal sperm count and testicular sorbitol dehydrogenase activity. Simultaneously, lactate dehydrogenase activity was reduced [16]. Normal sperm motility requires sources of energy that can produce adenosine triphosphate (ATP). Sperm contains two important monosaccharides, namely, sorbitol and fructose. Sorbitol dehydrogenase can convert sorbitol into fructose, which can be processed in sperm through the glycolytic pathway to produce ATP [41].

Exposure to stress during pregnancy is correlated with alterations in the reproductive system of the offspring. Adult male rats that experienced prenatal stress had the highest percentage of abnormal spermatozoa. They also displayed the lowest sperm motility and lower testis and epididymis weight than control rats [42]. Meanwhile, the rats treated with the daily supplementation of Tualang honey from their first day of pregnancy until delivery showed reduced detrimental stress effects. Furthermore, corticosterone levels were found to increase in humans and rats during pregnancy and in prenatally stressed male and female rats [43]. In general, the release of plasma corticosterone increases with the occurrence of stress and may cause major depression, which often occurs in pregnant women. Honey contains phenolic constituents, such as quercetin. In stressed rats, the administration of quercetin could significantly reduce the plasma corticosterone level [44].

The uniqueness of honey has attracted many researchers in the field of artificial insemination. The viscosity (resistance to flow) of honey increases with the reduction in temperature. As a result, honey thickens and condenses, and particle flow significantly decelerates. Honey undergoes a glass transition phase between −42 °C and −51 °C and forms a rubbery state within this temperature range. When the glass transition phase temperature decreases to less than −42 °C, honey turns into a glassy state with an amorphous property (noncrystalline) that prevents ice crystal formation [37]. Artificial insemination begins with semen collection, analysis, processing and cryopreservation. A high possibility exists for the semen to be exposed to thermal, mechanical, chemical osmotic and oxidative stress during cryopreservation. These elements can exert adverse effects on spermatozoa morphology and physiology. Therefore, the use of honey as a natural cryoprotectant has been widely studied and supported. Honey can act as a sperm protectant given its antibacterial activity and assists in spermatozoa survival by providing the primary sugar needed as an energy source.

A study conducted on the post-thawed semen of Arab stallions found that honey could be used as a remedy for cryopreservation injury [45]. This study showed that supplementation with honey as a semen extender significantly improved sperm motility, the viability index and membrane and acrosome integrities at zero to three hours of post-thawing. This finding was in line with the results of other studies conducted by [46,47,48] on cattle, sheep, common carp and goat, respectively. The supplementation of human semen with 10% pure honey in cryoprotectant medium resulted in a significant increase in the percentage of normal sperm morphology [49]. Additionally, a study conducted on the frozen-thawed sperm of common carp discovered that the addition of pine honey to the cryoprotectant positively affected sperm motility, fertilisation and eyeing and hatching success rates [48]. Notably, supplementation with rosemary honey combined with garlic and a skimmed milk-based extender reduced DNA fragmentation [47]. Table 1 summarises the role of honey in improving male fertility.

### 3.2. Role of Honey in Improving Postmenopausal Symptoms

Locals in Malaysia use honey mixed with egg yolk during the postpartum and postmenopausal periods. They believe that the nutrition provided by honey helps improve health and enhance vitality. Famous people in ancient times, namely, Aristotle (third century BC), Paulus Aegineta (seventh century AD) and Gilbertus Anglicus (13th century AD), stated that the average age of menopause for ancient women is 50.4 [50]. The mean age of menopause amongst Malaysian women was 48.7 years old in 1989 [51] and increased to 50.7 years old in 2003 [52]. Overall, the average age of menopause for women from ancient times is almost the same as those of women in modern times.

As women age, ovarian oestrogen production gradually declines. This phenomenon leads to a decline in reproduction capacity and various symptoms, such as vaginal dryness and genital atrophy, in women [53]. During the reproductive phase, the vaginal epithelium produces glycogen, which is anaerobically metabolised by commensal bacteria (lactobacilli) into lactic acid [54]. Lactic acid maintains the vaginal ecosystem by providing an acidic environment (low pH) that inhibits the growth of pathogenic or disease-causing bacteria. Furthermore, lactobacilli inhibit the growth of harmful bacteria because they produce H_2_O_2_. During the postmenopausal period, the production of oestrogen declines, lactobacilli become absent and the vaginal ecosystem becomes alkaline [55]. These effects promote favourable conditions for the growth of harmful organisms, particularly *E. coli*, thus resulting in bacterial vaginosis. Moreover, dyspareunia (painful intercourse) is one of the most frequently reported complaints of postmenopausal women due to the highly fragile, dry and easily traumatised condition of the vagina [56]. The size of the postmenopausal uterus tends to reduce to one-fourth of its earliest size. This effect is accompanied by uterine wall thinning, decreased vascularity and reduced function [57].

Hormone replacement therapy (HRT) has been widely used since 1960 to overcome the problems experienced by postmenopausal women. However, the long-term use of HRT increases the risk of breast cancer, endometrial cancer, thromboembolic events, vaginal bleeding, nausea, headaches and breast tenderness [58,59]. The reports on these issues have led to a search for an alternative to HRT. A previous study on a postmenopausal animal model (ovariectomised rats) found that the daily intake of Tualang honey for two weeks was significantly effective in protecting the vagina and uterus from atrophy [17]. Vacuolations of the vaginal epithelial cells, implying an increase in the mucopolysaccharide (glycogen) content, were also observed. These findings would benefit the use of honey as an alternative treatment for vaginal dryness in menopausal women. A similar result for the effects of Tualang honey was observed on the endometrium and myometrium of the uterus. However, some atrophic changes were still visible. The reduction in uterotrophic effects caused by Tualang honey in ovariectomised rats was important given that the risk of endometrial cancer is associated with oestrogen replacement therapy [60,61]. Therefore, honey can be recommended as an alternative therapy without significant side effects for HRT.

Phyto-oestrogen (oestrogen-like) compounds found in honey are responsible for the restoration of the atrophied uterus and vagina. A recent in vitro study involving MCF-7 cell cultures found that, at low concentrations, Manuka honey contributed oestrogenic effects by stimulating cell growth (oestrogen agonist). However, high concentrations of Manuka honey inhibited cell growth by exerting cytotoxic effects [62]. This finding was supported by a previous study, which claimed that phyto-oestrogens posed biphasic oestrogenic effects that are known as oestrogenic and antiestrogenic properties. These effects are dependent on the concentrations of circulating endogenous oestrogens [63]. Earlier studies on other beehive products found that propolis and royal jelly have potential oestrogenic effects on the uterus and vagina [21,22]. In 2002, Song et al. reported that propolis exerted a weak oestrogenic effect through its interaction with oestrogen receptors. Oestrogenic activities were examined through in vitro and in vivo assays, which included cell proliferation, oestrogen receptor binding, yeast-based steroid receptor (oestrogen, androgen and progesterone receptors), transcription and uterotrophic assays [22]. The in vivo uterine bioassay demonstrated that the increase in the tissue mass of immature female rats contributed to oestrogen-induced uterotrophic effects. Further studies on other beehive products found that some sterols were present in royal jelly [64]. These sterols accounted for oestrogenic activities via interaction amongst oestrogen receptors [21]. An in vivo study was performed to verify the in vitro results. Subsequently, royal jelly was proven to help increase the vascularity of the endothelial growth factor of the uterus of ovariectomised rats. Table 2 summarised the role of honey in improving postmenopausal symptoms.

### 3.3. Role of Honey against Toxicity in the Reproductive System

Reproductive toxicology can be defined as the study of biologically adverse effects on the male or female reproductive system resulting from exposure to environmental agents, particularly xeno-oestrogen compounds [65]. Toxicity can be due to the generation of free radicals associated with oxidative stress and inflammation in related organs and the endocrine system [66].

In the last few decades, the protective role of numerous herbal plants and natural products in preventing or reducing reproductive system toxicity has become highly acknowledged by a wide range of communities and cultures worldwide [67,68]. Bisphenol A (BPA) is one of the emerging environmental pollutants that affect health. It has been extensively studied for its toxic effects, especially its deleterious effects on the reproductive system, on many tissues of laboratory rodent animal models [69,70]. In general, numerous scientific studies have documented that BPA can induce the morphological and functional alteration of the male and female reproductive systems by influencing steroidogenic enzymes [71,72].

A 2014 study found that in BPA-exposed rats, Tualang honey, when consumed orally on a daily basis, could serve as an effective natural supplement to reduce the toxic effects of BPA by restoring the level of FSH and LH hormones [18,19]. A study on the protective effects of Tualang honey against the toxic effects of cadmium on the female reproductive system reported similar positive findings [73]. This result reflected the normalisation of GnRH in the brain. In BPA-exposed rats treated with Tualang honey, morphological abnormalities, such as the formation of large antral cystic-like follicles (anovulation follicles), the insufficiency of the corpora lutea and preantral follicles and the number of atretic follicles, were slightly reduced. In BPA-exposed rats, supplementation with honey significantly improved ovarian and uterine morphological abnormalities, reduced lipid peroxidation and normalised ERα, ERβ and C3 expression levels and distribution.

The protective effects of Tualang honey against BPA-induced female reproductive toxicity are attributed to the oestrogenic and antioxidant properties of specific bioactive molecules, namely, flavonol. Quercetin and kaempferol are the main naturally occurring flavonols that share structural similarities with 17β-oestradiol. Therefore, the potential oestrogenic effects of these compounds are comparable with those of other xeno-oestrogens, such as BPA [74]. A recent study by Henderson et al. showed that Manuka honey exerted oestrogenic effects on MCF-7 breast cancer cells [62]. Notably, another study demonstrated that honey showed inhibitory effects on the breast cancer cell lines MCF-7 and MCF-10A by inducing cytotoxicity and genotoxicity [75]. Although these studies illustrated the stimulatory and inhibitory effects of honey on cancerous cells, further studies are required to understand the mechanisms and effective concentrations of honey.

CS is one of the most important toxicants that pose a high risk to male reproductive function. It contains a complex mixture of more than 7000 chemical compounds, such as nicotine, carbon monoxide and other substances. These substances are highly toxic to the human organism and include many reactive oxygen species (ROS) [76,77,78]. Clinical studies have demonstrated that CS is strongly associated with reproductive decline due to several factors, such as impaired sperm production [79], motility [80] and ED [81]. Animal model studies found that several adverse effects of CS include reductions in fertilising potential and the generation of oxidative stress in penile tissue [82]. In CS-exposed rats, the administration of Tualang honey resulted in an improvement in daily sperm production and percentage of motile sperm, increased spermatid and sperm counts, and reduced abnormal sperm production and the toxic effects of CS on spermatogenesis. These results were achieved through increases in testosterone levels [83]

A similar study also found significant improvements in several histopathological parameters that were associated with the reduction in the percentage of tubules with germ cell loss, the increment in the diameter of seminiferous tubules and height of seminiferous epithelial cells, and the restoration of Leydig cell count [23]. These findings might suggest that honey poses a protective effect against oxidative stress in rat testis. Lipid peroxidation (TBARS), glutathione peroxidase (GPx), total antioxidant status (TAS) and superoxide dismutase (SOD) and catalase (CAT) activities in rats exposed to CS and treated with honey were assessed in vitro. The results of this assessment suggest that honey’s antioxidant activity plays a significant role in reducing the adverse effects of CS because it could counteract the increase in ROS by decreasing TBARS and GPx. Simultaneously, it increases antioxidant enzyme activities, as shown by the results for SOD, CAT and TAS. Table 3 summarised the role of honey against toxicity in the reproductive system.

### 3.4. The Role of Honey as a Vulvovaginal Candidiasis (VVC) Treatment

VVC, which is also known as yeast infection, is one of the gynaecological disorders that affect women’s lives. This condition is due to the disruption of commensal organisms, including *Lactobacillus acidophilus,* in the normal vaginal environment; this disruption may accelerate the growth of pathogens, such as *Candida* species [84,85,86]. On the basis of supporting evidence presented in epidemiological studies by [87] in the United States, *Candida albicans* is believed to be the primary cause of VVC. Specifically, 90% of the pathogens that cause VVC are represented by *C. albicans*, whereas the rest are non-albican species [88]. *Candida glabrata* has been found to cause the recurrence of VVC [89]. Individuals who face the problem of VVC often experience the disruption of physical activities, low self-confidence and difficulties in carrying out an intimate relationship, wherein introital dyspareunia may occur [90].

Previous studies have found that a relatively high number of women suffer from VVC. A total of 75% of women become infected with VVC at least once in their lifetime; 40 to 50% of patients experience the recurrence of VVC and 5% suffer from recurrence [91,92]. Denning et al. (2018) reported that this infection is experienced by individuals aged 25 to 34 years old and has an annual global prevalence of 9% [90].

Vaginal colonisation by *Candida* species leads to pruritus, irritation, vulval and vaginal erythema, oedema, and in the most extreme cases, severely malodorous vaginal discharge [93]. This issue may result from multiple factors, including the use of broad-spectrum antibiotics, the intake of oral contraceptives, insufficient immune cells, obesity, high doses of oral oestrogen [94] and the absence of *Lactobacillus* necessary for acidity regulation to prevent fungal growth.

Azole antifungal agents were proposed as a treatment for VVC in 1969 [95]. Various formulations of effective azole antifungal remedies, such as imidazoles (e.g., butoconazole, clotrimazole and miconazole) and triazoles (e.g., fluconazole and terconazole) are available. Widely available remedies include over-the-counter products and prescription drugs in topical and oral forms. However, the prolonged treatment and consumption of over-the-counter products may contribute to the development of strains with high-level azole resistance [96]. The solution to this issue has been widely discussed in studies because women are either triggered by the desire to prevent the potential side effects of pharmaceutical drugs or are becoming increasingly interested in applying natural methods in their health care [86].

Honey, a natural product and a therapeutic and antimicrobial agent, has been recognised since ancient times as a traditional medicine. The antimicrobial activity of honey appears to be associated with H_2_O_2_ production through the glucose oxidase enzyme, acidity, bee defensin-1 and osmolarity [88]. Honey is considered as an option for VVC treatment because it has been determined to inhibit the growth of *C. albicans* without affecting *Lactobacillus*. *Lactobacillus*—the normal vaginal flora regulator—produces lactic acid, which maintains the vaginal pH at normal levels, and inhibits bacterial adherence to vaginal epithelial cells.

Banaeian-Borujeni et al. (2013) reported that 33% honey exerted a strong inhibitory effect on *C. albicans* [97]. This finding was in line with that of Al-Waili (2005), who found that honey collected from the United Arab Emirates could significantly inhibit the growth of various pathogenic fungi, including *C. albicans*, at concentrations of 30 to 100% [98]. This point was reinforced by a recent finding showing that honey could relieve vaginitis symptoms without adverse effects [20]. Similarly, some other studies have reported on the effectiveness of honey in inhibiting the growth of *C. albicans* in culture media [97,99,100].

Several studies have compared the antimicrobial effects of different types of honey. Sayadi et al. (2015) compared selected Malaysian honey types—Gelam, Tualang, Nenas and Acacia—with Manuka honey and found that all the tested honey types (except for Gelam honey) had inhibitory effects against the tested microorganisms [101]. Manuka honey displayed the most potent antifungal activity against all strains, including *C. albicans*, which was found to be the most susceptible fungi. Another study compared honey from different parts of Turkey, specifically, Izmir, Sivas, Afyon and Mugla. Izmir honey exerted more significant antimicrobial effects on *Staphylococcus aureus*, *E. coli* and *Pseudomonas aeruginosa* than on *C. albicans*. Mugla honey presented inhibitory effects against *C. albicans* [102]. The difference between these results might be due to the diversity of the plant origin of the honey. This diversity led to the production of a variety of honey compounds. Overall, the differences between the various obtained findings could be ascribed to the different antibiotic activities performed by different types of honey.

The use of honey in combination with other substances as remedies to VVC was also studied. Yoghurt is typically used as an alternative treatment for vaginal infections due to its low price and therapeutic effectiveness. *Lactobacillus* can be found in yoghurt. It can control the normal vaginal environment and inhibit the growth of fungi when used directly to treat infections. The use of the combination of honey with yoghurt as vaginal cream was compared with that of clotrimazole in nonpregnant women with VVC infections [91]. This combination was also compared with itraconazole in pregnant women who were infected with VVC [88]. Both studies indicated that, compared with the local antifungal agent, the mixture of yoghurt and honey not only showed similar therapeutic effects but was also more effective in relieving the symptoms of VVC (itching, irritation, dysuria, dyspareunia and discharge). Moreover, a study on pregnant women found that the mixture of honey and yoghurt resulted in an improvement of 87.8% in the symptoms of VVC, whereas itraconazole consumption resulted in an improvement of 72.3%. Therefore, honey could be recommended as an alternative to antifungal agents due to its safety of use and availability at a low price.

One of the major antimicrobial factors of honey is the generation of H_2_O_2_. Honey mainly generates H_2_O_2_ when diluted because of the activation of glucose oxidase, which oxidises glucose into gluconic acid and H_2_O_2_ [103,104]. Glucose oxidase is an enzyme found in the nectar added by the bees. Although this enzyme is not activated in mature honey, it regains its activity when honey is diluted. Matured honey provides sufficient protection through its high osmotic pressure and acidity properties. According to Irish et al. (2006), the H_2_O_2_-type of honey has a considerable antifungal effect [105].

The osmolarity effects caused by high sugar contents are also be considered as beneficial antimicrobial factors of honey [106]. Banaeian-Borujeni et al. (2013) claimed that honey consists of a high concentration of carbohydrates (82%) and contains a low amount of water [97]. This composition contributes to the inhibition of microorganisms, especially bacteria that cannot adapt to high osmotic pressure during dehydration. The acidity properties of honey can also inhibit bacteria. The pH value of honey ranges from 3.2 to 4.5 and is low (acidic) and unsuitable for bacterial growth, which requires a minimum pH level of 7.2 to 7.4.

Overall, honey is a natural substance that is recommended for the treatment of VVC infection given its availability, convenience and cost-effectiveness. It is also a natural component that does not require sterilisation and does not cause considerable side effects, making it the most interesting natural remedy. Table 4 summarised the role of honey as a vulvovaginal candidiasis treatment.

## 4. Conclusions

Honey is a well-known natural product with medicinal properties that can cure various health problems given its high nutritional value and antioxidant properties. In reproductive health, honey can improve infertility, protect the postmenopausal reproductive tract, prevent toxic effects, maintain sperm quality by restoring testosterone levels and treat VVC infections. Many benefits of honey remain unrevealed by studies, thus providing researchers with the opportunity to investigate the potential of honey and contribute to the scientific research regarding this remarkable God-gifted product.

## Figures and Tables

**Table 1:** Summary of the role of honey in improving male fertility.

Type of Honey	Type of Study	Findings	Reference
Tualang Honey	In vivo	1.2 g/kg/day Tualang honey significantly increased epididymal sperm count without affecting spermatid count and reproductive hormones.	[25]
Persian honey	In vivo	Honey decreases cellular damage and apoptosis during testicular injury, with significant protective effects on reproductive hormone production.	[40]
Tualang Honey	In vivo	Supplementation of honey during pregnancy seems to reduce the adverse effects of restraint stress on reproductive organs weight and sperm parameters in male rat offspring.	[42]
Honeybee	Human Study	Supplementation of 10% honeybee resulted in a significantly increased percentage of normal sperm morphology.	[49]

**Table 2:** Summary of the roles of honey in improving postmenopausal symptoms.

Type of Honey	Type of Study	Findings	Reference
Tualang honey	In vivo	Tualang honey was shown to have beneficial effects on menopausal (ovariectomised) rats by preventing uterine atrophy, increased bone density and suppression of increased body weight.	[17]
Royal jelly from *Apis mellifera L*	In vivo and in vitro	Royal jelly has estrogenic activities through interaction with oestrogen receptors, followed by gene expression changes and cellular functions.	[21]

**Table 3:** Summary of the roles of honey against toxicity in the reproductive system.

Type of Honey	Type of Study	Findings	Reference
Tualang Honey	In vivo	Honey supplementation has a protective effect against damage and oxidative stress induced by chronic smoking in rat testis via significantly reduced histological changes and TBARS level, increased TAS level, and restored activities of GPx, SOD and CAT considerably.	[23]
Tualang honey	In vivo	Tualang honey reduces the toxic effects of BPA by restoring FSH and LH hormones, reducing morphological abnormalities of the ovarian follicles and improving the normal oestrous cycle.	[19]
Tualang honey	In vivo	Tualang honey has protective effects against cadmium-induced ovarian toxicity by reducing morphological abnormalities, restoring the normal levels of gonadotropin hormones and stabilising the equilibrium levels of lipid peroxidation and antioxidant enzyme in ovaries of rats.	[73]
Tualang honey	In vivo	Tualang honey improved daily sperm production and the percentage of motile sperm, increased spermatid and sperm counts, and reduced abnormal sperm production and the toxic effects of CS on spermatogenesis.	[83]
Tualang honey	In vitro	Tualang honey enhanced 4-hydroxytamoxifen-induced cytotoxicity and DNA damage in breast cancer cells while protecting the non-cancerous cells.	[75]

**Table 4:** Summary of the roles of honey as a vulvovaginal candidiasis treatment.

Type of Honey	Type of Study	Findings	Reference
Honey cream	Human study	Honey contributed to relieving and recovering vaginitis symptoms with no adverse effects were reported by patients	[20]
Honey from the Chahar Mahal and Bakhtiari province, Iran	In vitro	Honey prevented the growth of *C. albicans* considerably only at an 80% concentration but did not affect the normal vaginal flora, *Lactobacillus*.	[97]
Honey from UAE	In vitro	Honey and honey mixture inhibited the growth of *C. albicans* and *S. aureus*.	[98]
Honey from Chahar Mahal Bakhtiari province, Iran	In vitro	Honey (80%) inhibited the growth of *C. albicans*, but it did not affect the *Lactobacillus,* which can be used to treat Candidiasis without changed variation in the vaginal flora.	[99]
Variety of honey from Algeria	In vitro	All varieties of honey were effective against *C. albicans.*	[99]
Manuka honey, Gelam honey, Nenas honey, Tualang honey and Acacia honey	In vitro	All tested Malaysian honeys except Gelam showed antifungal activity against all species analysed, with the minimum inhibitory concentration ranging from 25 (*v*/*v*) to 50% (*v*/*v*). In comparison, the minimum inhibitory concentration of Manuka honey ranged from 21 to 53% (*v*/*v*).	[101]

## Data Availability

Not applicable.
